# The learning curve in robotic assisted knee arthroplasty is flattened by the presence of a surgeon experienced with robotic assisted surgery

**DOI:** 10.1007/s00167-022-07048-6

**Published:** 2022-07-21

**Authors:** Clemens Schopper, Philipp Proier, Matthias Luger, Tobias Gotterbarm, Antonio Klasan

**Affiliations:** 1 Department for Orthopedics and Traumatology, Kepler University Hospital GmbH, Krankenhausstrasse 9, 4020 Linz, Austria; 2 Johannes Kepler University Linz, Altenberger Strasse 69, 4040 Linz, Austria

**Keywords:** Robotics, Robotically assisted surgery, Total knee arthroplasty, Unicompartmental knee arthroplasty, Learning curve

## Abstract

**Purpose:**

The purpose of this study was to investigate the learning curve associated with robotic assisted knee arthroplasty (RAS KA). Therefore, the evaluation of the influence of an experienced surgeon on the overall team performance of three surgeons regarding the learning curve in RAS KA was investigated. It was hypothesized that the presence of an experienced surgeon flattens the learning curve and that there was no inflection point for the learning curve of the surgical team.

**Methods:**

Fifty-five cases consisting of 31 total knee arthroplasties (TKA) and 24 unicompartmental arthroplasties (UKA) performed by three surgeons during 2021 were prospectively investigated. Single surgeon and team performance for operation time learning curve and inflection points were investigated using cumulative sum analysis (CUSUM).

**Results:**

A downward trend line for individual surgeons and the team performance regarding the operation time learning curve was observed. No inflexion point was observed for the overall team performance regarding TKA and UKA. The surgeon that performed all cases with the assistance of the experienced surgeon had significantly shorter surgical times than the surgeon that only occasionally received assistance from the experienced surgeon (*p* = 0.004 TKA; *p* = 0.002 UKA).

**Conclusion:**

The presence of an experienced surgeon in robotically assisted knee arthroplasty can flatten the learning curve of the surgical team formerly unexperienced in robotic assisted systems. Manufacturers should provide expanded support during initial cases in centres without previous experience to robotic assisted knee arthroplasty.

**Level of evidence:**

III.

## Introduction

Learning curves are well known in medical education and surgical training [9]. Robotic assisted surgery (RAS) is becoming increasingly popular in total knee arthroplasty (TKA) and unicompartmental knee arthroplasty (UKA) [4]. RAS has demonstrated more accurate implant positioning in vivo and in vitro when compared to the conventional instruments [1, 4, 8].

For both TKA and UKA, RAS has shown no learning curve with the implant positioning but a significant learning curve with the workflow and instrumentation [3, 4, 12]. Surgical time flattens out effect after 5–10 cases and, in most studies, reaches a steady value after 8 cases in image-based RAS for UKA and TKA [4, 6, 11, 17, 18, 20, 22]. RAS offers a good opportunity for low-volume arthroplasty surgeons to achieve high levels of implant position accuracy in TKA and UKA [10].

Literature to date mainly reflects single surgeon performance, or a group's performance where the whole group goes through the learning curve simultaneously [19, 20]. Individual surgeon characteristics, such as experience with arthroplasty or experience with digital technology as are, however, difficult to quantify and some studies show a learning curve up to 40 cases [21]. Since each centre and thus, surgeons within that centre, start with RAS simultaneously, it remains unclear if the presence of a RAS-experienced surgeon influences the learning curve of the surgical team including RAS-inexperienced surgeons. Therefore, this study sought to answer the following questions: (1) What is the influence of a RAS experienced surgeon on the learning curve for RAS TKA and UKA of the surgical team? and (2) Is there an inflection point in the learning curve of the surgical team? It was hypothesized that the presence of an experienced surgeon flattens the learning curve and that there was no inflection point for the learning curve of the surgical team.

## Methods

This study was performed at a tertiary arthroplasty referral centre. Ethics board approval was obtained prior to commencement of the study (1146/2021). Consecutive patients with end stage knee osteoarthritis undergoing primary RAS UKA and RAS TKA between June 2021 and October 2021 were included in this study. Indications for RAS UKA were based on the criteria by Hamilton et al. [7], whereas all other patients received a RAS TKA. Age, gender, BMI and ASA were collected. The indications for UKA and TKA remained unchanged with the commencement of the study. The standard implants prior to implementation of RAS were CI (conventionally instrumented) Oxford Phase 3 mobile-bearing UKA (Zimmer Biomet, Warsaw, IN, U.S.) and CI Persona TKA (Zimmer Biomet). Three surgeons were included in the study and have completed the same 2-day clinical application certification: first year post residency surgeon with no prior exposure to CAS (computer assisted surgery) or RAS (Surgeon 1, PP); an 11 years post residency surgeon with no prior exposure to CAS or RAS (Surgeon 2, TG); a dual-fellowship trained surgeon with significant prior exposure to both CAS and RAS (Surgeon 3, AK).

RAS UKA (MAKO Rio, Stryker, Kalamazoo, MI, U.S.) was performed using a standardized work-flow [16] with the application of both the saw and the burr, using proprietary implants (Restoris, Stryker). RAS TKA was performed using a standardized work-flow [16]. Implants (cemented Triathlon, Stryker) were positioned by applying functional alignment strategy [2] aiming to respect the anatomical position of the femur as much as possible. Surgeon 3 routinely resurfaced the patella, Surgeon 2 did not resurface, Surgeon 1 selectively resurfaced.

Surgeons 1 and 3 immediately switched all knee arthroplasty to RAS, whereas surgeon 2 switched to 20% caseload. Surgeons 1 and 3 performed all cases together, surgeon 2 performed 50% of the cases with surgeon 1 and 50% with surgeon 3. Six scrub nurses were involved in the implementation phase, without being controlled for in the present study. A total of 55 cases were performed during the study period, 31 TKAs and 24 UKAs. There were no differences in demographic data between surgeons Table 1.

**Table 1:** Demographic data

	Surgeon 1	Surgeon 2	Surgeon 3	*p* value
Age	69.6 ± 9.2	72.8 ± 8.0	69.8 ± 8.7	0.578
Female%	38.4%	36.4%	31.3%	0.780
BMI	31.4 ± 4.5	30.2 ± 4.9	31.2 ± 5.8	0.793
ASA 2%	75.0%	72.7%	68.8%	0.820
*n* cases (UKA)	28 (11)	11 (5)	16 (8)	

### Outcome measures

**Fig. 1:**
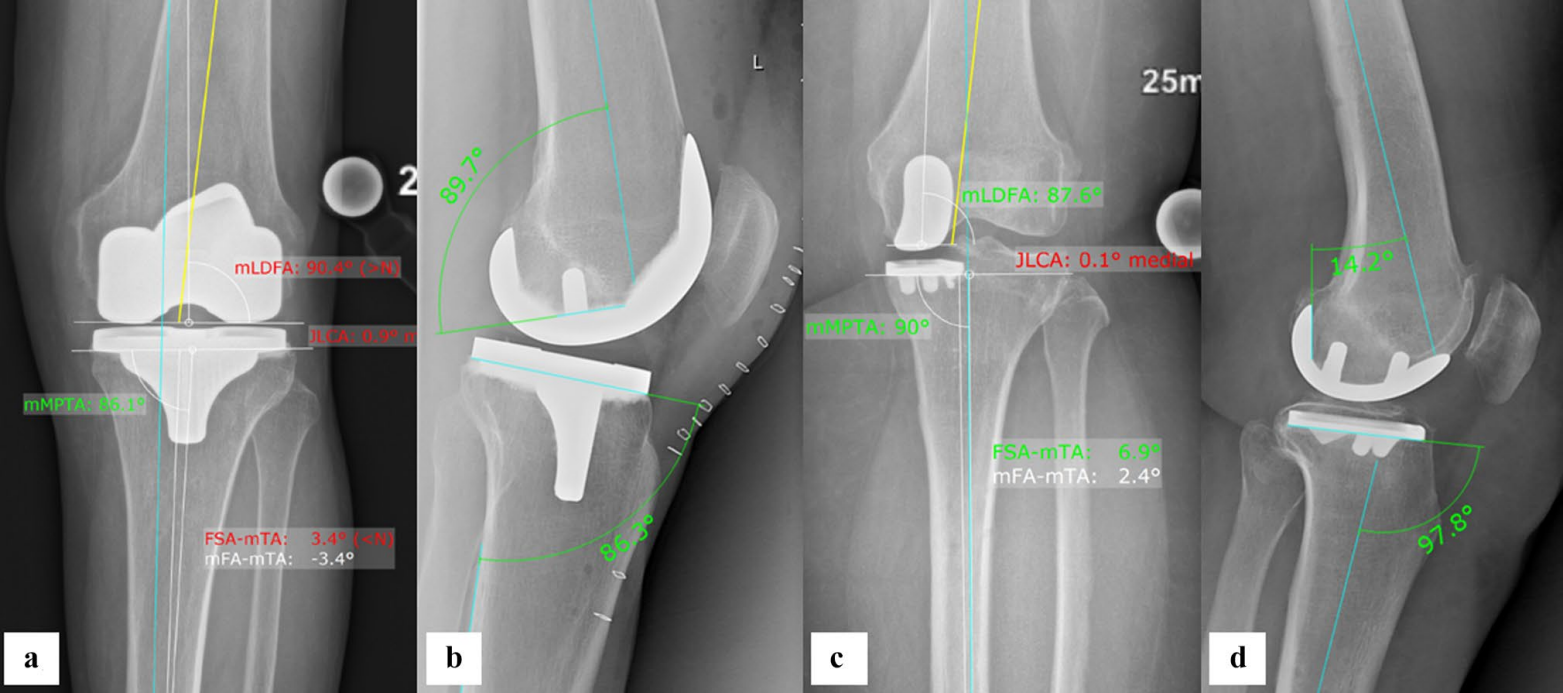
**A**–**D** The details for implant positioning are shown for each TKA (**A**, **B**) and UKA (**C**, **D**) in AP and lateral view x rays. *mLDFA* mechanical lateral distal femoral angel, *mMPTA* mechanical medial proximal tibial angle, *JLCA* joint line conversion angle, *FSA-mTA* femoral shift axis-mechanical tibial axis, *mFA-mTA* mechanical femoral axis-mechanical tibial axis

The primary outcome measure was surgical time, measured from initial incision until final wound closure. The surgical time has been further broken down into following parts of the procedure: surgical approach, bone referencing, balancing, bone preparation, trialling, final implementation and closure. Secondary outcome measure was the accuracy of implant positioning. Patients in both treatment groups underwent pre- and postoperative anteroposterior knee, lateral knee and full leg-length standing radiographs. Two independent observers (CS and ML) determined the accuracy of implant positioning by comparing the value achieved intraoperatively to the planned value in the corresponding postoperative radiograph. Measurements were performed using MediCad (Medicad Hectec, Altdorf, Germany). Agreement between the observers was investigated by interclass correlation coefficient (ICC). Femoral and tibial axes were used as reference markers to assess accuracy of all positioning and measure alignment in degrees [1], with a tolerance of 1.0°. Coronal [13, 15] and sagittal alignment [5] were measured on AP and lateral views, Fig. 1A–D.

Finally, all patients were followed-up at week 2, 6 and 12 postoperatively by the independent observers for potential complications and adverse events.

### Statistical analysis

Normality distribution was analyzed using the Shapiro–Wilk test. Normally distributed continuous data are presented with mean ± standard deviation (SD), non-normally distributed data with median [IQR]. Continuous data were compared using ANOVA and Mann–Whitney *U* Test, depending on the distribution. CUSUM (cumulative sum) analysis was performed as previously described [12]. Due to the variability of previous experience between surgeons, surgeon 3 data was used as a baseline and comparator for surgical time, but was not used as the target time in the CUSUM analysis. A prospective analysis of consecutive cases has been performed. A *post-hoc power* analysis with a beta of 0.9 and an alpha of 0.05 revealed 4 cases per surgeon to detect a difference in surgical times for TKA and 6 cases per surgeon to detect a difference for UKA. IBM SPSS statistics v27 (Armonk, NY, U.S.) was used for statistical analysis. Significance was set at p < 0.05.

## Results

### Surgical time

Only surgeon 2 had longer mean surgical time than surgeons 1 and 3, Table 2.

**Table 2:** Surgical times

	Surgeon 1	Surgeon 2	Surgeon 3	*p* value
Surgical time TKA (mins)	73.6 (± 10.9)	94.3 (± 14.3)	70.1 (± 5.6)	0.004
Surgical time UKA (mins)	64.8 (± 9.7)	77.2 (± 3.1)	60.2 (± 5.3)	0.002

**Fig. 2:**
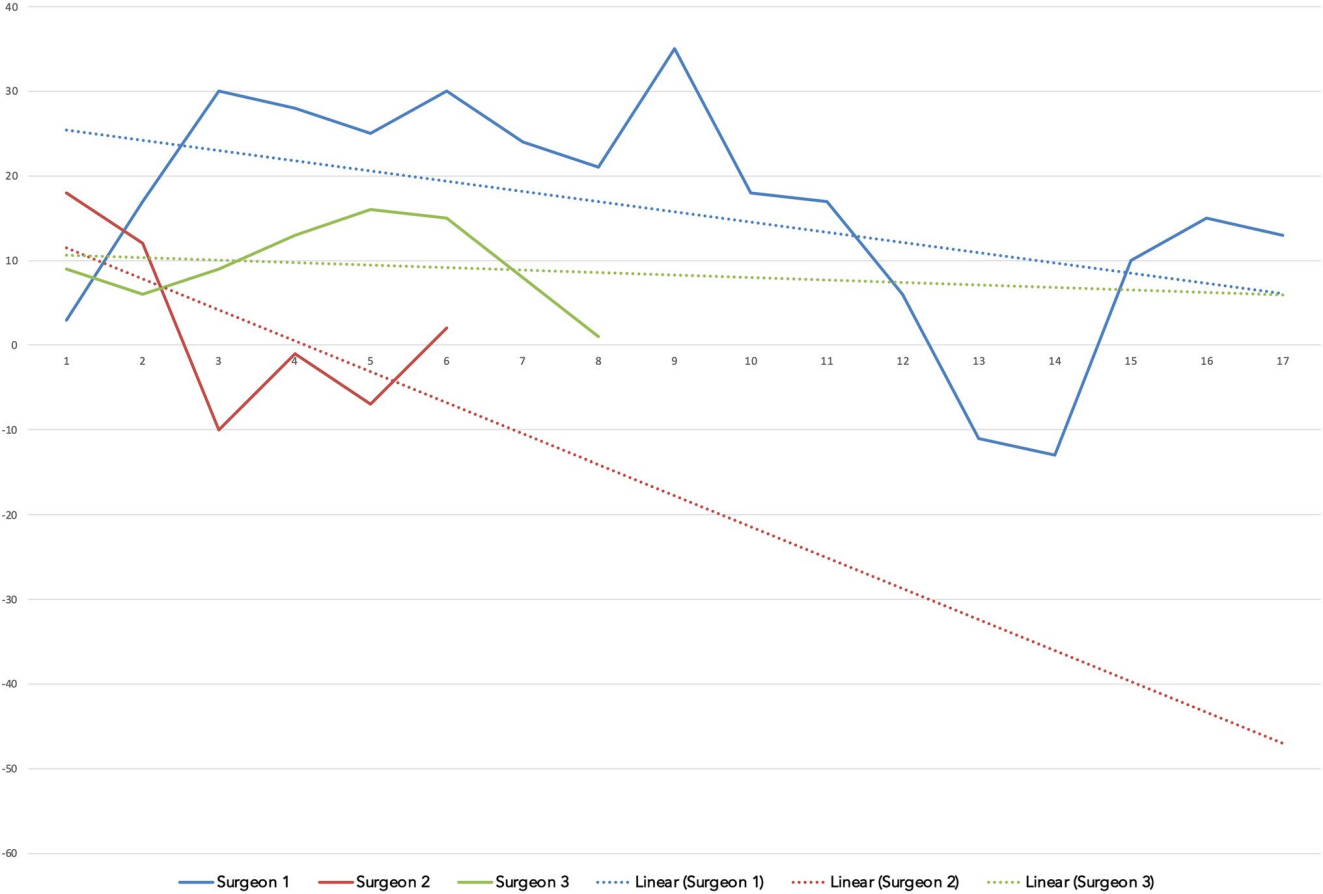
Single surgeon CUSUM analysis for the operation time of the TKA cases is shown. *X* axis displays the running case number, *Y* axis displays the CUSUM value

CUSUM analysis of TKA cases, Fig. 2, demonstrated no inflection point for surgeons 2 and 3 and three peak values in the surgical time learning curve for surgeon 1. A maximum CUSUM value of 35 and a downward trendline for all surgeons was observed.

**Fig. 3:**
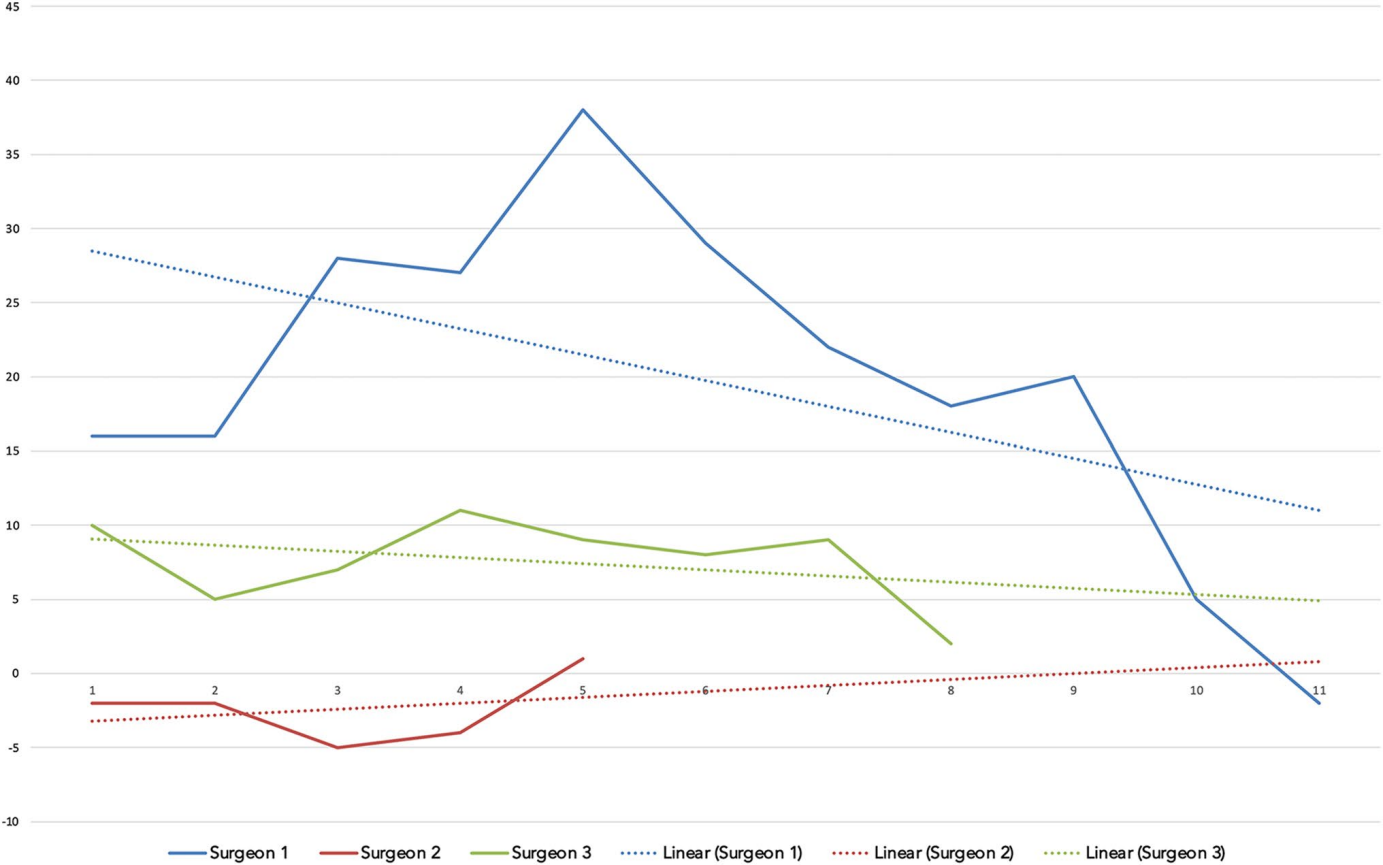
Single surgeon CUSUM analysis for the operation time of the UKA cases is shown. *X* axis displays the running case number, *Y* axis displays the CUSUM value

CUSUM analysis of UKA cases, Fig. 3, demonstrated an inflection point at case 5 for surgeon 1, but only with a maximum CUSUM value of 35.

**Fig. 4:**
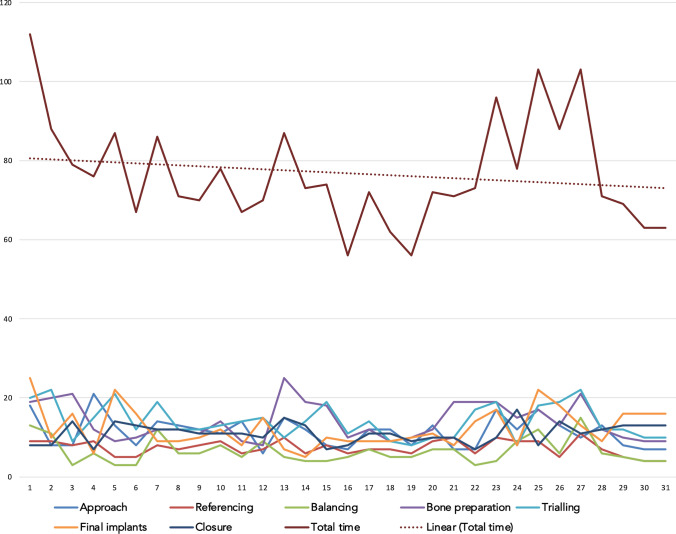
Team surgical time analysis for TKA cases is shown in the head and the contribution of surgical parts towards total time and per case as well in the bottom. *X* axis displays the running case number, *Y* axis displays the time in minutes

**Fig. 5:**
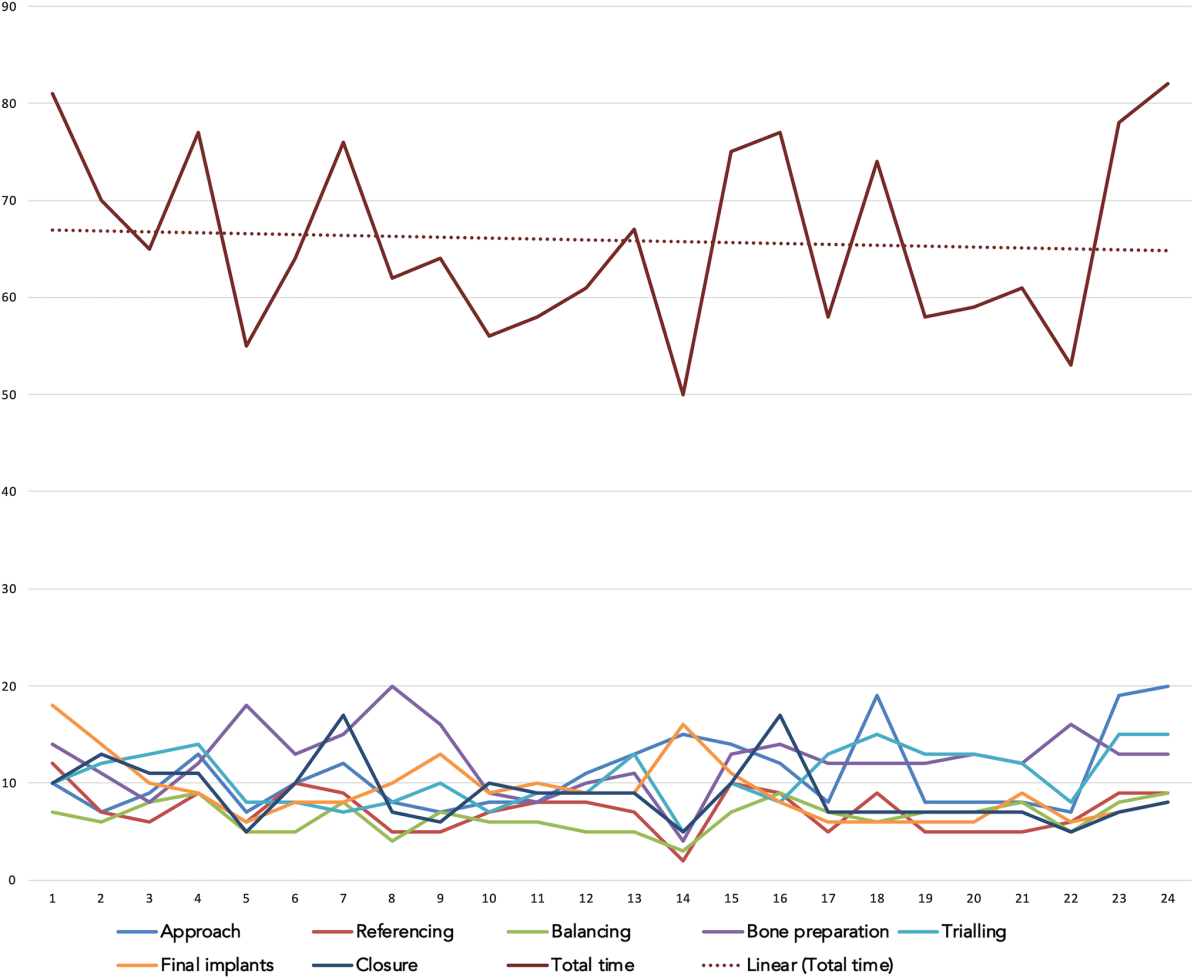
Team surgical time analysis for UKA cases is shown in the head and the contribution of surgical parts towards total time and per case as well in the bottom. *X* axis displays the running case number, *Y* axis displays the time in minutes

Mean surgical times of TKA and UKA, Figs. 4 and 5, reveal a downward slope from the first case.

### Implant positioning

There were no outliers in implant positioning between the intraoperative values and postoperative values obtained from radiographs. ICC for radiographic measurements was 0.94.

Two patients had an adverse event. One female after TKA sustained a fall at week 8, rupturing the patellar tendon and puncturing the skin horizontally, thus exposing the joint. After an unsuccessful DAIR (Debridement, Antibiotics and Implant retention) and patellar tendon reconstruction, a first stage revision with patellar tendon re-reconstruction was performed. There were no issues at follow-up at week 2 and 6. Second patient fell during week 3, opening up her scar proximally. A DAIR has been performed, at 12 weeks after revision surgery, there were no further issues.

## Discussion

The most important finding of this study was that the presence of an RAS experienced surgeon flattens the learning curve of an RAS unexperienced surgical team.

Kayani et al. previously demonstrated that six cases were needed until an inflexion point is achieved [12]. In the present study, an inflection point could be observed at 5 cases for UKA and at 9 cases for TKA respectively. There were major differences for the 2 surgeons, with one of the different variables being the presence of the experienced surgeon providing assistance. As Vermue et al. previously noted [21], some surgeon specific variables are difficult to quantify, but a significant variable here was the RAS-experienced surgeon providing assistance. This study nevertheless does demonstrate a clear learning effect reflected in surgical time reduction, but these are lower than in comparative studies [4, 6, 11, 12]. Learning curves are observed in other new technologies, not just RAS. Gharaibeh et al. [6] analyzed intercompartmental pressure differences after TKA between the lateral and the medial compartment using a newly introduced sensor-guided assessment technique for soft tissue balancing. They compared 2 groups of each 45 cases displaying the balancing for the first 45 against the second 45 cases. In the first group 10 cases were identified as unbalanced whereas every case in the second group was well balanced. A number needed to treat of 30 was identified at which the inflection point occurred for the learning curve for proper balancing with this technique whereas no effect was displayed for the surgical time [6]. The described effect of improvement comparing starting cases to end stage cases is also known from other works [14]. Even longer learning curve effects compared to these findings have been observed. Vermue et al. [21] report up to 35 and 43 cases until an inflection point was reached. The authors believe that inconsistencies in the surgical team are responsible for these differences [21]. This effect was not found in the present study, although a total of 6 scrub nurses were included. No inflection points occurred in the analysis of the team performance. This demonstrates the beneficiary effect of the experienced surgeon´s presence on the overall team performance. The importance of the presence of an experienced surgeon is also underlined by the findings of Vermue et al. [21]. Although consistency of the surgical team was not assured for the scrub nurse in our setting, the described negative effect on the surgical time performance was not observed in the present study [21]. Thus, the presence of an experienced surgeon can neutralize a potential negative effect of team inconsistency on the surgical time performance. Interestingly, the downward slope for RAS UKA was flatter than for RAS TKA. This might be connected to the fact that one of the 2 RAS-unexperienced surgeons has had significant experience in classic jig-based UKA. Similar effect was observed by Kayani et al. since different pre-existing grades of experience for classic jig-based KA may lead to different learning curves for RAS KA [12, 20]. Similarly, Vermue et al. indicate that different learning curves depend on the individual, pre-existing surgical experience in classic jig-based TKA [21]. No outliers in alignment were observed in this study, which corresponds to previous results of both RAS-TKA and RAS-UKA [4, 12].

The primary limitation is a small sample size of 55 cases, divided between 3 surgeons, as well as the absence of a STAI score evaluation. The results, however, demonstrate the investigated effect even with this sample size. The individual surgical characteristics, such as previous surgical experience including number of cases performed have not been quantified. As pointed out by Vermue et al., in a study with six surgeons, quantification of all potential variables is almost impossible and is therefore disregarded in the present study. One surgeon only performed 5 UKA, which is not enough to assess the learning curve, which might take as long as 40 cases, as per Vermue, however, the initial times are significantly longer than for the other inexperienced surgeon that always had the experienced surgeon as the assistant. Patella resurfacing philosophy between surgeons in the present study has been different, however, the changes in time would not alter the results observed, since there is no learning curve for the conventional patella and the less experienced RAS surgeons either did not use the patella or only occasionally used the patella. Furthermore, no RAS currently offers a patella application, which could be assessed as a part of the learning curve. The follow-up for a clinical study is short, 90 days, but is longer than in comparable studies. Another limitation finally is found in the circumstances set by the COVID-19 pandemic probably inherent to every elective surgical discipline: Reduced elective operative capacity and surgical cases may not only have led to a significantly reduced case load but also to a disturbance of the development of the learning curve for RAS KA in this case series. Finally, a control group is missing in this study so that the learning curve behaviour can only be described in the manner of tendencies, although a true control group would almost be impossible because each surgeon can only undergo one learning curve, in this case 1 surgeon with assistance of an experienced surgeon and 1 surgeon with occasional assistance, with different results. Patient reported outcomes are not reported, since the study only has a short follow-up and clinical outcomes are not the primary outcome of the study. Lack of adverse events and radiographic evidence of excellent implant position serve as a proxy for outcomes.

## Conclusion

The presence of an experienced surgeon in robotically assisted knee arthroplasty can flatten learning curve of the surgical team formerly unexperienced in robotic assisted systems. Manufacturers should provide expanded support during initial cases in centres without previous experience to robotic assisted knee arthroplasty.

## Abbreviations

RAS KA: Robotic assisted knee arthroplasty
TKA: Total knee arthroplasties
UKA: Unicompartmental arthroplasties
CUSUM: Cumulative sum analysis
THA: Total hip arthroplasty
CAS: Computer-assisted surgery
CI: Conventionally instrumented
ICC: Interclass correlation coefficient
SD: Standard deviation
IQR: Interquartile range
